# Comparison of In Vitro Multiple Physiological Activities of Cys–Tyr–Gly–Ser–Arg (CYGSR) Linear and Cyclic Peptides and Analysis Based on Molecular Docking

**DOI:** 10.3390/biom16010126

**Published:** 2026-01-12

**Authors:** Ga-Hyun Kim, Jeong-Eun Bang, Bo-Mi Kim

**Affiliations:** Department of Chemical Engineering, Wonkwang University, Iksan-si 54538, Jeollabuk-do, Republic of Korea; gahyun1214@wku.ac.kr (G.-H.K.); wjddms4403@wku.ac.kr (J.-E.B.)

**Keywords:** linear peptide, cyclic peptide, CR5 peptide, DPPH radical scavenging, tyrosinase inhibition, MMP-1 inhibition, molecular docking

## Abstract

Peptide cyclization is a strategy to improve biological stability and functional activity, but direct comparison between linear and cyclic peptides with the same sequence is still limited. In this study, linear (L-CR5) and cyclic (C-CR5) forms were synthesized, and biological functions such as antioxidant, whitening, and anti-wrinkle activity were compared and evaluated. C-CR5 showed about 22.3 times of DPPH radical scavenging activity, which was significantly stronger than L-CR5, and tyrosinase inhibition increased rapidly in C-CR5 to reach inhibition of 95% or more, whereas L-CR5 showed only moderate activity in the same range (about 6.5 times). MMP-1 expression in the evaluation of anti-wrinkle activity did not show a decreasing trend in L-CR5 at all, while C-CR5 showed an anti-wrinkle effect, which was reduced by about 92.8% at 400 μg/mL. As a result of molecular docking analysis, C-CR5 exhibited lower MolDock scores than L-CR5 toward both tyrosinase and MMP-1, indicating a potentially higher binding affinity and improved binding stability. This is expected to be due to reduced structural flexibility and optimized residue directions (especially Tyr and Arg). These results indicate that peptide cyclization is an example of enhanced functional bioactivity of CYGSR and provides a positive case for the structure–activity relationship.

## 1. Introduction

Linear peptides offer advantages such as facile synthesis and flexible sequence design. However, they are structurally more flexible and therefore susceptible to enzymatic degradation [1,2]. This high degree of conformational variability reduces their stability and persistence in biological environments, ultimately leading to diminished bioactivity and overall efficacy [3,4]. Peptide cyclization has gained attention as a strategy to overcome these limitations [5].

Peptides have garnered significant attention as bioactive materials owing to their structural diversity and biocompatibility, which enable a wide range of physiological activities [6,7]. In particular, peptide-based research related to skin antioxidant, whitening, and anti-wrinkle effects has been actively explored [8,9,10,11]. Cyclic peptides form conformationally constrained structures through linkages between the N-terminus and C-terminus or via side-chain cyclization. Such conformational restriction enhances resistance to enzymatic degradation, improves stability, and increases binding affinity, while also strengthening hydrogen bonding, π–π interactions, and electron donating interactions at target active sites [12,13]. Therefore, cyclization is considered an important design strategy for enhancing the bioactivity of peptides [14].

CR5 (Cys–Tyr–Gly–Ser–Arg; CYGSR), in particular, has previously been reported as a cyclic peptide exhibiting strong multifunctional activities related to skin, including antioxidant, whitening, and anti-wrinkle effects [15]. Although several studies have compared linear and cyclic peptides, direct comparisons using identical amino acid sequences remain limited. Consequently, additional investigations are needed to accumulate structure–activity relationship (SAR) data to better elucidate how cyclization influences biological function [16,17,18,19,20,21].

The CYGSR sequence used in this study was finally selected as the sequence (CYGSR) that exhibited multifunctional efficacy after screening among candidate sequences redesigned with reference to previous studies on collagen-derived peptides [22]. According to the literature, cysteine and tyrosine have been reported to be associated with antioxidant activity through radical scavenging mediated by thiol and phenolic groups, respectively [23,24,25,26]. Glycine, a major amino acid component of collagen, is known to be involved in maintaining the structural stability of the extracellular matrix [27]. In addition, serine is a key component of the natural moisturizing factor (NMF) and is associated with water binding capacity, while arginine has been reported to exhibit biological activities related to cellular activation and collagen synthesis [28,29]. Based on these literature findings, CR5 was designed as a candidate peptide incorporating residues potentially relevant to skin physiological activities.

The mechanisms through which cyclic peptides exert their biological activities have been reported to be closely associated with their structural features [30,31]. For whitening activity, the conformationally constrained three dimensional structure of cyclic peptides allows for more complementary spatial alignment within the active site of tyrosinase, leading to improved inhibitory effects on melanogenesis [32,33]. Regarding anti-wrinkle activity, cyclic peptides generally possess higher stability and selectivity than their linear counterparts, resulting in increased resistance to enzymatic degradation (e.g., MMP-1) and prolonged persistence in the skin. Consequently, they can more effectively suppress MMP-1-mediated collagen degradation and help maintain skin elasticity by preventing the loss of extracellular matrix components, particularly type I procollagen [34,35].

In addition, these structural effects can be quantitatively assessed through molecular docking analysis, which enables detailed comparison of differences in binding affinity and interaction stability between the linear and cyclic forms. Cyclization has been reported to stabilize binding energies toward target proteins such as tyrosinase and MMP-1, promoting stronger interactions by optimizing intermolecular distances and orientations [36,37]. The cyclic backbone reduces the number of freely rotatable bonds, thereby increasing conformational stability and enhancing complementarity to the active site, ultimately leading to a more stable binding pose [13,14,33,35,36,38,39]. Notably, peptide sequences containing Tyr and Arg residues may exhibit enhanced interactions with the active sites of tyrosinase or MMP-1 in their cyclic form, as these residues can be positioned more favorably within the constrained ring conformation [40].

Studies comparing linear and cyclic peptides have been conducted for a variety of biological targets. However, systematic principles—defined as general structure-based design rules—explaining how cyclization influences changes in biological activity, reductions in binding energy, improvements in docking scores, and stabilization of interaction networks have not yet been clearly established [41,42]. Furthermore, data directly comparing linear and cyclic peptides with identical amino acid sequences remain extremely limited, indicating the need for extensive comparative analyses to identify consistent structural features that govern the influence of cyclization on structure–activity relationships [18,43].

Therefore, in this study, we performed a comparative in vitro evaluation of three biological activities, along with molecular docking analysis, using a linear form (Linear CYGSR, L-CR5) and a cyclic form (Cyclic CYGSR, C-CR5) that share the identical amino acid sequence (CYGSR). Through this approach, we aimed to elucidate how cyclization influences target-protein binding affinity and skin-related bioactivities from the perspective of the SAR.

## 2. Materials and Methods

### 2.1. Materials

#### 2.1.1. Chemicals

The reagents used for peptide synthesis and chemical analyses were purchased from Sigma-Aldrich (St. Louis, MO, USA) or Tokyo Chemical Industry (Tokyo, Japan), and the amino acids were obtained from GL Biochem (Shanghai, China).

#### 2.1.2. Cell

The mouse-derived macrophage cell line RAW 264.7 and the human dermal fibroblast cell line CCD-986sk were obtained from the Korean Cell Line Bank (KCLB, Seoul, Republic of Korea).

#### 2.1.3. Assay Kits

Cell viability was measured using the EZ-CYTOX reagent (EZ-1000; Daeil Lab Service, Seoul, Republic of Korea). Protein content was determined using the Pierce BCA Protein Assay Kit (23227; Thermo Fisher Scientific, Waltham, MA, USA). MMP-1 secretion was analyzed using the Human Total MMP-1 Quantikine ELISA kit (DMP100; R&D Systems, Minneapolis, MN, USA). For protein quantification, an albumin standard (23209; Thermo Fisher Scientific) was used as the reference.

#### 2.1.4. Instruments

The synthesized peptides were analyzed using a High-Performance Liquid Chromatography (HPLC) system with a Waters e2695 separation module equipped with a 2998 photodiode array (PDA) detector (Waters Corp., Milford, MA, USA). Purification was performed using a Waters 2545 quaternary pump module equipped with a 2998 PDA detector (Waters Corp.). Peptide synthesis data acquisition and processing were carried out using Empower 3.0 software (Waters Corp.).

In addition, a low-temperature laboratory chamber (JSS-700C, JS Research Inc., Gongju, Republic of Korea), a microplate multi-mode reader (Synergy HTX, BioTek Instruments, Winooski, VT, USA), and a microbalance (ARG4202, OHAUS Corp., Parsippany, NJ, USA) were used in this study.

### 2.2. Methods

#### 2.2.1. Synthesis of L-CR5

In this study, the peptide was synthesized using solid-phase peptide synthesis (SPPS). 2-Chlorotrityl chloride resin (CTC, 0.50 g, 1.00 mmol/g) was swirled in dichloromethane (DCM, 5.80 mL) for 30 min.

The first amino acid, Fmoc-Arg(Pbf)-OH (2.00 equivalents, 0.60 g), and *N*,*N*-diisopropylethylamine (DIEA, 5.00 equivalents, 0.40 mL) were added in DCM (5.80 mL), and the coupling reaction was carried out for 4 h. To deactivate unreacted sites, capping was performed for 10 min using a DCM:MeOH:DIEA (15:3:2, *v*/*v*/*v*) solution. The Fmoc protecting group was then removed using 20% piperidine in *N*,*N*-dimethylformamide (DMF).

In the second coupling reaction, Fmoc-Ser(tBu)-OH (2.00 equivalents, 0.40 g) was reacted with *N*-hydroxybenzotriazole (HOBt), DIEA (5.00 equivalents, 0.40 mL), and *N*,*N*-diisopropylcarbodiimide (DIC, 3.00 equivalents, 0.20 mL) in DMF (4.00 mL) for 4 h. Completion of the coupling reaction was confirmed by the Kaiser test. Unreacted sites were capped for 10 min using a DMF: acetic anhydride:DIEA (8:1:1, *v*/*v*/*v*) solution, and Fmoc deprotection was carried out with 20% piperidine/DMF for 30 min.

In the third coupling reaction, Fmoc-Gly-OH (2.00 equivalents, 0.30 g), in the fourth, Fmoc-Tyr(tBu)-OH (2.00 equivalents, 0.50 g), and in the fifth, Fmoc-Cys(Trt)-OH (2.00 equivalents, 0.60 g) were coupled under the same conditions as previously described.

#### 2.2.2. Cleavage of L-CR5

The synthesized peptide was cleaved from the resin using a cleavage cocktail composed of trifluoroacetic acid (TFA), triisopropylsilane (TIS), and H_2_O (95:2.5:2.5, *v*/*v*/*v*). After adding 12 mL of the cleavage solution, the mixture was stirred for 1 h. The resin was then filtered off, and cold isopropyl ether (IPE, 66 mL) was added to the filtrate to precipitate the peptide as a white solid.

The product was obtained as a white solid with a yield of 68%.

#### 2.2.3. Cyclization of L-CR5

The linear peptide L-CR5 was converted into its cyclic form through the intramolecular formation of an amide linkage between the N-terminal amine and the C-terminal carboxyl group. To initiate the reaction, lyophilized L-CR5(1.00 mmol) was dissolved in DMF to obtain a 1.00 mM solution. Subsequently, HOBt (2.5 equivalents), *N*-ethyl-*N*′-(3-dimethylaminopropyl) carbodiimide hydrochloride (EDC·HCl, 2.5 equivalents), and DIPEA (5.0 equivalents) were introduced sequentially at 0 °C under continuous stirring. The mixture was then allowed to warm to room temperature and maintained under a nitrogen atmosphere. Reaction progress was assessed by HPLC at 4 h intervals. After about 12 h, the cyclization was completed, and the reaction was quenched by the addition of cold IPE, which facilitated peptide precipitation. The precipitated material was extracted with ethyl acetate (three times), and the combined organic fractions were dried over anhydrous Na_2_SO_4_ and concentrated under reduced pressure. The crude cyclized peptide (C-CR5) was further purified using reversed-phase HPLC.

The product was obtained as a white solid with a yield of 31%.

#### 2.2.4. HPLC Purification and Analysis

Peptide purification was carried out using a YMC-Pack ODS-A-HG C18 reversed-phase column (250 × 20 mm, 10 µm, 30 nm, YMC, Kyoto, Japan). The mobile phases consisted of solvent A (water containing 0.1% TFA) and solvent B (acetonitrile containing 0.1% TFA). A 50% gradient was applied over 45 min, and the flow rate was set to 20 mL/min. The separated peptide peaks were collected, lyophilized, and obtained as powdered peptides.

Chromatograms obtained before and after purification of L-CR5 are provided in Supplementary Figure S4.

HPLC analysis was performed using a Waters system with UV detection at 210 nm and 230 nm. The same instrument used for purification was employed for analysis, and an analytical XBridge C18 reversed-phase column (4.6 × 250 mm, 5 µm, Waters Corp., USA) was used. The mobile phases consisted of solvent A (water containing 0.1% TFA) and solvent B (acetonitrile containing 0.1% TFA). The gradient was set to 65% solvent B at 35 min, with a flow rate of 1.0 mL/min and an injection volume of 20 µL. The purity was estimated to be >95% based on HPLC peak area.

#### 2.2.5. MALDI-TOF MS

Since L-CR5 and C-CR5 are newly synthesized cyclic peptides, it was essential to verify that their observed *m*/*z* values corresponded to their theoretical monoisotopic masses (584.65 Da and 566.64 Da, respectively) prior to biological evaluation. MALDI-TOF mass spectrometry was therefore performed using an Autoflex maX MALDI-TOF/TOF mass spectrometer equipped with a SmartBeam II laser (355 nm; Bruker Daltonics, Bremen, Germany) at the High-Tech Materials Analysis Core Facility.

A saturated solution of α-cyano-4-hydroxycinnamic acid prepared in TA50 (acetonitrile:0.1% TFA in water = 1:1, *v*/*v*) was used as the matrix. L-CR5 and C-CR5 samples were individually dissolved in 0.1% TFA, mixed with the matrix solution at a 1:1 ratio, spotted onto a MALDI target plate, dried at room temperature, and subjected to analysis.

Mass spectra were acquired in positive-ion reflectron mode under the following conditions: laser power, 35–40%; repetition rate, 2000 Hz; ion source 1, 19 kV; ion source 2, 16.8 kV; and pulsed ion extraction time, 110 ns. A total of 500 laser shots were accumulated within the mass range of 300–4000 Da. Data processing was performed using FlexAnalysis software (version 3.4, Bruker Daltonics).

External calibration was conducted using the Bruker Peptide Calibration Standard, which includes Angiotensin II, Angiotensin I, Substance P, Bombesin, ACTH (1–17), ACTH (18–39), and Somatostatin-28.

#### 2.2.6. Cell Culture

The mouse-derived macrophage cell line RAW 264.7 was cultured in a 100 mm cell culture dish using DMEM supplemented with 10% fetal bovine serum (FBS) and 1% penicillin/streptomycin in a 5% CO_2_ incubator. Cells that reached confluence were passaged using a cell scraper.

CCD-986sk cells were cultured in IMDM medium containing 10% FBS and 1% P/S in 150 mm cell culture dishes and maintained in a 5% CO_2_ incubator. When the cells reached confluence, they were passaged using a cell scraper.

#### 2.2.7. Cell Viability

Dehydrogenases present in the mitochondria of viable cells convert the tetrazolium salt (WST) into a colored product called formazan. RAW 264.7 cells were seeded at a density of 1 × 10^4^ cells/well in a 96-well cell culture plate and cultured for 24 h in DMEM supplemented with 10% fetal bovine serum (FBS) and 1% penicillin/streptomycin. After incubation, the medium was replaced with DMEM containing the test samples at various concentrations, followed by an additional 24 h of incubation. EZ-CYTOX reagent (DoGenBio, Seoul, Republic of Korea) was then added to the cells, and absorbance was measured at 450 nm. Cell viability was calculated according to Equation (1).

CCD-986sk cells (3 × 10^3^ cells/well) were seeded in a 96-well cell culture plate and cultured for 24 h in IMDM medium supplemented with 10% FBS and 1% P/S. The cells were then starved in FBS-free IMDM medium containing 1% P/S for more than 6 h. Subsequently, the medium was replaced with IMDM containing the samples. Each sample was dissolved in distilled water to prepare a stock solution, diluted to the desired concentrations, and then further diluted in IMDM to achieve the final treatment concentrations. The sample containing medium was added to the cells, followed by incubation for 48 h. After the treatment period, IMDM medium containing 10% EZ-CYTOX solution was added and incubated for 2 h. Absorbance was measured at 450 nm using a microplate reader, and cell viability was calculated according to Equation (1).

Cell viability (%):

(1) (A_sample_/A_control_) × 100

#### 2.2.8. DPPH Assay

The synthesized peptides and ascorbic acid (positive control) were prepared at various concentrations (Based on the results of the preceding preliminary experiment, DPPH radical scavenging ability was evaluated in the range of 25–400 μg/mL of L-CR5 and in the range of 3.13–200 μg/mL considering the higher efficacy of C-CR5). To perform the assay, 12.5 μL of each sample was added to 50 μL of ethanol, followed by the addition of 62.5 μL of 0.1 mM 2,2-diphenyl-1-picrylhydrazyl (DPPH). The mixture was incubated at 4 °C in the dark for 30 min, and the absorbance was measured at 520 nm. The concentration of remaining DPPH radicals was calculated according to Equation (2).

Free radical scavenging activity (%):

(2) 100 − (b − b′/a − a) × 100

a: absorbance of the control after reaction

b: absorbance of the sample after reaction

a′, b′: absorbance measured by replacing 0.1 mM DPPH with buffer

#### 2.2.9. Tyrosinase Inhibition Assay

Tyrosinase inhibitory activity was evaluated by measuring the absorbance using mushroom tyrosinase. The synthesized peptides and the positive control (β-arbutin) were diluted to various concentrations. Each reaction mixture was prepared by adding 10 µL of the sample, 10 µL of mushroom tyrosinase (1500 U/mL), and 20 µL of L-tyrosine (1.5 mM) to 110 µL of 0.1 M sodium phosphate buffer (pH 6.5). The mixed solution was incubated at 37 °C for 10 min, and the absorbance was measured at 490 nm. Tyrosinase inhibitory activity was calculated according to Equation (3).

Tyrosinase inhibition (%):

(3) 100 − (b − b′/a − a′) × 100

a: Absorbance of the control solution after the reaction;

b: Absorbance of the sample solution after the reaction;

a′, b′: Absorbance measured by replacing mushroom tyrosinase with buffer instead of the enzyme.

#### 2.2.10. MMP-1 Expression Inhibition ELISA Assay

CCD-986 cells were seeded at a density of 1.5 × 10^4^ cells/well in a 24-well cell culture dish and cultured for 24 h in IMDM medium supplemented with 10% FBS and 1% penicillin-streptomycin. The cells were starved in serum-free IMDM for 6 h. After washing with 1 mL of DPBS, the cells were exposed to UV at 5 J/cm^2^. The samples were then diluted in culture medium and treated for 48 h, and TGF-β1 (10 ng/mL) was used as the positive control. After 48 h, the culture supernatants were collected and analyzed by ELISA, and the MMP-1 inhibition rate was calculated according to Equation (4).

MMP-1 Expression Inhibition (%):

(4) 100 − (b/a) × 100

a: corrected expression level of the untreated group;

b: corrected expression level of the treated group.

#### 2.2.11. Determination of Protein Content

Protein content was measured using the Pierce BCA Protein Assay Kit. After adding 200 µL of the Pierce BCA Protein Assay Reagent mixture to 20 µL of the collected culture medium, the mixture was incubated at 37 °C for 30 min, and the absorbance was measured at 570 nm. Protein content was determined using an albumin standard as the reference.

#### 2.2.12. Data Analysis and Statistical Processing

The statistical significance of the bioactivity assay results was evaluated using GraphPad Prism 8 software (GraphPad Software, San Diego, CA, USA). One-way and two-way ANOVA were performed, followed by Dunnett’s post hoc test, and differences were considered statistically significant when the *p*-value was less than 0.05.

#### 2.2.13. Molecular Docking Simulation

To perform the molecular docking simulation, the three-dimensional structures of the peptide compounds L-CR5 and C-CR5 were generated using Chem3D Ultra software (ver. 8.0). Prior to docking, both peptides were protonated to reflect their stable charge states at physiological pH (7.4), followed by an energy minimization process to obtain optimized low-energy conformations. The protein structures of mushroom tyrosinase (PDB ID: 2Y9X; https://www.rcsb.org/structure/2Y9X, accessed on 18 November 2025) and MMP-1 (PDB ID: 1HFC; https://www.rcsb.org/structure/1HFC, accessed on 18 November 2025) were downloaded from the RCSB Protein Data Bank (RCSB PDB, https://www.rcsb.org/, accessed on 18 November 2025). Before docking, co-crystallized ligands and water molecules were removed, whereas metal ions essential for protein function were preserved (tyrosinase: Cu(II); MMP-1: Zn(II)/Ca(II)).

Docking simulations were conducted using Molegro Virtual Docker 6.0 (MVD) to predict the binding modes between the peptides and the target proteins. The grid box was centered on the active site, with coordinates set to X = −5.39, Y = −0.97, Z = −67.76 Å for tyrosinase and X = 14.54, Y = 30.08, Z = 24.38 Å for MMP-1. The search radius was configured to 15 Å to allow sufficient exploration of the ligand-binding region.

The docking scores reported in this study were obtained from the top-ranked docking pose with the lowest MolDock score, which was selected as the representative structure. The MolDock Score is a value that estimates the free energy change (ΔG), and a pose with the lowest binding energy (kcal/mol) is selected as the optimal structure as a negative value increases. The main interaction between L-CR5 and C-CR5 and the active site residues (hydrogen bonds, hydrophobicity) of the protein was visually identified using the Discovery Studio Visualizer (version 2025).

## 3. Results

### 3.1. Structures of L-CR5 and C-CR5

**Figure 1:**
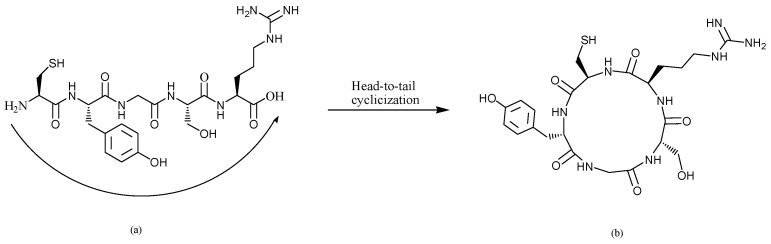
Chemical structures of (**a**) the linear peptide L-CR5 and (**b**) the cyclic peptide C-CR5 obtained through head-to-tail cyclization.

In this study, the linear peptide L-CR5 was first synthesized using solid-phase peptide synthesis (SPPS) under optimized coupling conditions designed to maximize reaction efficiency. The resulting linear peptide was then cyclized through a liquid-phase cyclization method to obtain the cyclic peptide C-CR5. A structural comparison of the linear and cyclic forms is presented in Figure 1. Unlike the relatively flexible linear peptide, the cyclic form adopts a more conformationally restricted and structurally stabilized ring architecture due to its enhanced steric constraints.

### 3.2. Molecular Weight Determination of the Cyclic Peptide (C-CR5)

MALDI-TOF MS analysis was performed to confirm the molecular weight of C-CR5, which is essential for the success or failure of the synthesis of cyclic peptides. Since one molecule of water (18.01 Da) is removed from the linear precursor L-CR5 (theoretical single isotope mass of 584.65 Da) during the cyclization process, the theoretical mass of cyclic C-CR5 is calculated as 566.64 Da. In the measured MALDI-TOF spectrum, C-CR5 showed the main peak at *m*/*z* 566.1, which corresponds to [M + H]^+^ ion that matches the theoretical value very well. In addition, the peak near *m*/*z* 587.9 is judged to be a [M + Na]^+^ sodium ion, and the peak near about *m*/*z* 1130 is judged to be a dimer ion in the form of [2M + H]^+^ These results confirm that the linear L-CR5 was successfully cyclized to produce C-CR5 having the designed molecular weight, and the original data are attached to Supplementary Figure S1.

### 3.3. Cytotoxicity Evaluation of L-CR5 and C-CR5

**Figure 2:**
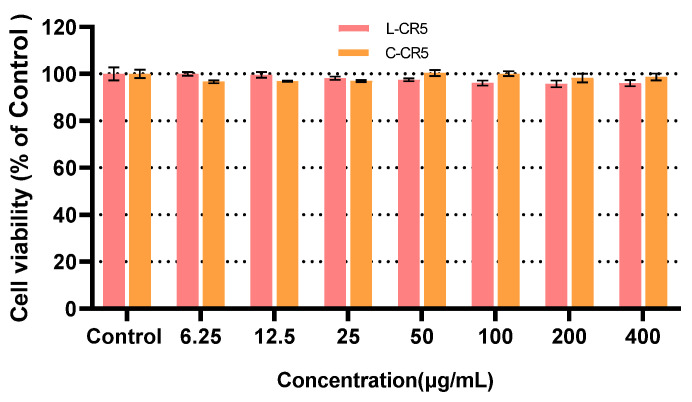
Cell viability of RAW 264.7 macrophages treated with L-CR5 and C-CR5.

As a result of treating the RAW 264.7 macrophages with L-CR5 and C-CR5 at a concentration of 6.25–400 μg/mL, cell viability was maintained at 95% or more in all treatment groups. As shown in Figure 2, both peptides did not show significant cytotoxicity in the entire concentration range, and stable cell survival was confirmed. In particular, it was confirmed that both L-CR5 and C-CR5 maintained a normal cell survival level even at a high concentration of 400 μg/mL, and thus did not cause toxicity in RAW 264.7 cells. These results suggest that the two peptides have sufficient cell safety to be used in subsequent physiological activity evaluation experiments.

### 3.4. Comparison of DPPH Radical Scavenging Activity Between L-CR5 and C-CR5

**Figure 3:**
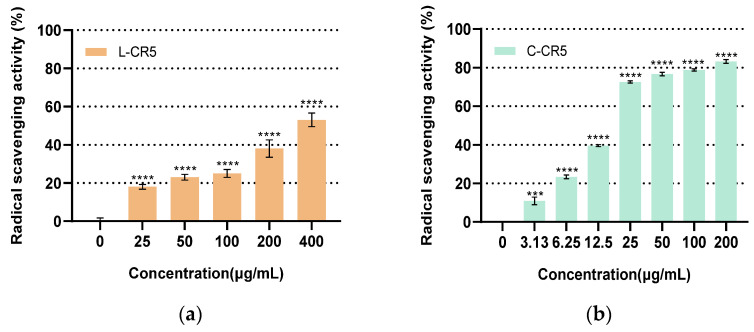
Comparison of DPPH radical scavenging activity between L-CR5 and C-CR5: (**a**) DPPH treated with L-CR5 (IC_50_ = 377.83 ± 34.32 μg/mL; (646.25 ± 58.70 μM), *n* = 3); (**b**) DPPH treated with C-CR5 (IC_50_ = 16.92 ± 0.47 μg/mL; (29.86 ± 0.83 μM), *n* = 3). *** *p* < 0.001, **** *p* < 0.0001.

As a result of comparing the DPPH radical scavenging ability of L-CR5 (Figure 3a) and C-CR5 (Figure 3b), the activity of both peptides increased in a concentration-dependent manner, but the cyclic C-CR5 showed remarkably superior antioxidant efficacy than linear L-CR5 in the entire concentration. The scavenging ability of L-CR5 gradually increased from 18% to 53% in the range of 25–400 μg/mL, whereas C-CR5 showed very high activity from 73% to 83% under the same concentration condition, and thus a difference in efficacy of at least 2.5–3.5 times or more was confirmed. Particularly, at the intermediate concentration (50–100 μg/mL), the L-CR5 showed a significant difference of 23–25% and the C-CR5 77–79% (*p* < 0.001). This trend was also consistent in the IC_50_ analysis. The IC_50_ of L-CR5 was calculated as 377.83 ± 34.32 μg/mL (646.25 ± 58.70 μM), whereas the IC_50_ of C-CR5 was found to be 16.92 ± 0.47 μg/mL(29.86 ± 0.83 μM), and thus C-CR5 showed about 22.3 times stronger antioxidant activity than that of L-CR5. This suggests that even based on the same sequence, structural stability and electron donation arrangement are improved by cyclization, which can dramatically enhance antioxidant function. The antioxidant assay was conducted using ascorbic acid as the positive control, and the dose–response plots along with the calculated IC_50_ values for the control group are presented in Supplementary Figure S2.

### 3.5. Comparison of Tyrosinase Inhibitory Activity of L-CR5 and C-CR5

**Figure 4:**
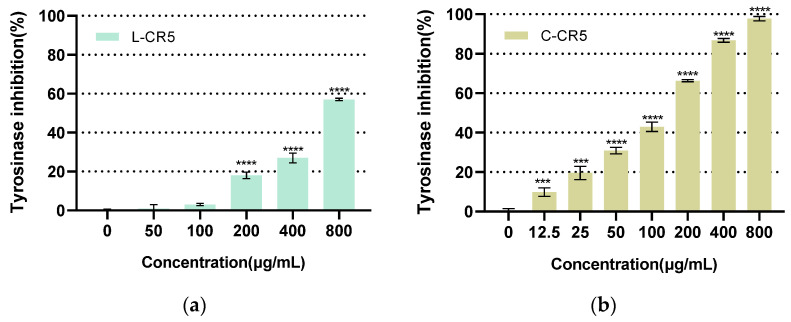
Comparison of tyrosinase inhibitory activity between L-CR5 and C-CR5: (**a**) tyrosinase treated with L-CR5 (IC_50_ = 680.63 ± 15.68 μg/mL; (1164.0 ± 27.3 μM), *n* = 3) (**b**) tyrosinase treated with C-CR5 (IC_50_ = 104.2 ± 5.3 μg/mL; (184.0 ± 9.4 μM), *n* = 3). *** *p* < 0.001, **** *p* < 0.0001.

As a result of comparing the tyrosinase inhibitory activity of L-CR5 (Figure 4a) and C-CR5 (Figure 4b), both peptides showed increased concentration-dependent activity, but C-CR5 showed significantly better inhibitory activity than L-CR5 in the entire concentration section. In the range of 50–800 μg/mL, the tyrosinase inhibitory rate of L-CR5 gradually increased from 1.16% to 57.35%, while C-CR5 showed a rapid increase from 30.91% to 97.79% in the same concentration section. In particular, at the intermediate concentration of 50 μg/mL and 100 μg/mL, C-CR5 showed about 26.6 times and about 9.9 times higher inhibitory activity, respectively, thereby maximizing the difference in efficacy between the two peptides. Although the evaluation concentration ranges of the two peptides were not completely consistent, the relative superiority and inferiority of the efficacy were judged by comparing the IC_50_ values calculated based on each concentration–response curve, and the IC_50_ of C-CR5 supported the higher efficacy than L-CR5.

This trend was also consistent in IC_50_ analysis. The IC_50_ of L-CR5 was calculated as 680.63 ± 15.68 μg/mL (1164.0 ± 27.3 μM), while the IC_50_ of C-CR5 was identified as 104.2 ± 5.3 μg/mL (184.0 ± 9.4 μM), indicating that C-CR5 had about 6.5 times more potent antioxidant activity compared to L-CR5. The tyrosinase inhibition assay was performed using arbutin as the positive control, and the corresponding dose–response curves and IC_50_ values are provided in Supplementary Figure S3.

### 3.6. Comparison of MMP-1 Expression of L-CR5 and C-CR5

**Figure 5:**
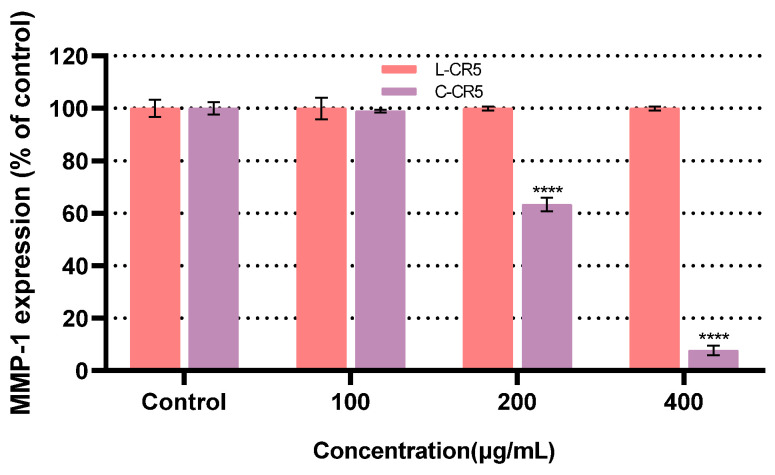
Comparison of MMP-1 expression between L-CR5 and C-CR5: MMP-1 expression was evaluated in cells treated with L-CR5 (*n* = 3) and C-CR5 (*n* = 5). **** *p* < 0.0001.

Comparing the inhibitory effects of L-CR5 and C-CR5 on MMP-1 expression after UV stimulation, both peptides maintained similar levels of MMP-1 expression at low concentrations (25–100 μg/mL), but the inhibitory effects were markedly different (Figure 5). L-CR5 showed expression levels of 99–100% in the 25–100 μg/mL range, had little tendency to decrease, and no significant inhibitory effect was observed at 100.80% even at 200 and 400 μg/mL. On the other hand, the expression level of C-CR5 decreased sharply from 200 μg/mL to 63.31% and decreased to 7.78% at 400 μg/mL, showing almost complete inhibitory effects. In particular, the inhibitory level of MMP-1 expression of C-CR5 at the highest concentration (400 μg/mL) was about 14 times stronger than that of L-CR5, allowing the introduction of ring structures even with the same sequence to achieve a control result that significantly enhanced the inhibitory effect of MMP-1.

In the MMP-1 assay, TGF-β1 (10 ng/mL) was used as a fixed-dose positive control, which induced MMP-1 expression to 49.34% relative to the untreated control group. This value was used as the reference level for evaluating the inhibitory effects of L-CR5 and C-CR5.

### 3.7. Docking of L-CR5 and C-CR5 Molecules with Target Proteins (Tyrosinase and MMP-1)

In this study, molecular docking simulations were performed to investigate the differences in binding characteristics between the linear (L-CR5) and cyclic (C-CR5) peptides, which share an identical amino acid sequence, toward the target proteins tyrosinase and MMP-1. The 3D structures of L-CR5 and C-CR5 used for molecular docking are provided in the support Figure S5. The analysis focused on comparing how cyclization influences binding affinity and interaction patterns within the active sites. The resulting MolDock Score, H-bond Energy, and Rerank Score values are summarized in Table 1.

As a result of tyrosinase docking, C-CR5 showed a lower MolDock Score than L-CR5, showing a relatively high binding affinity. The MolDock Score of C-CR5 was −110.02 kcal/mol, which has a lower energy value compared to the MolDock Score of −75.36 kcal/mol of L-CR5. This suggests that C-CR5 binds to the active site of tyrosinase much more stably. Hydrogen bonds also confirmed that C-CR5 had a negative value of −14.81 kcal/mol, which was higher than −10.54 kcal/mol of L-CR5, and thus cyclization increased the number of hydrogen bonds contributing to binding stability.

A similar trend was observed in the docking results for MMP-1. The MolDock Score of L-CR5 was −69.10 kcal/mol, and the hydrogen bond energy was −3.24 kcal/mol, whereas C-CR5 showed −99.64 kcal/mol and −9.20 kcal/mol, respectively. This indicates that C-CR5 exhibits more favorable binding characteristics at the MMP-1 active site compared with L-CR5. This suggests that C-CR5 has stronger binding interactions within the MMP-1 active site.

To characterize the molecular differences in binding behavior between the linear L-CR5 and cyclic C-CR5 peptides, their interactions with tyrosinase and MMP-1 were examined using 2D interaction diagrams.

**Figure 6:**
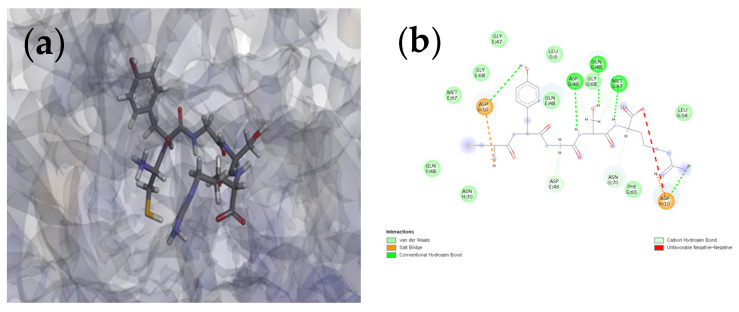

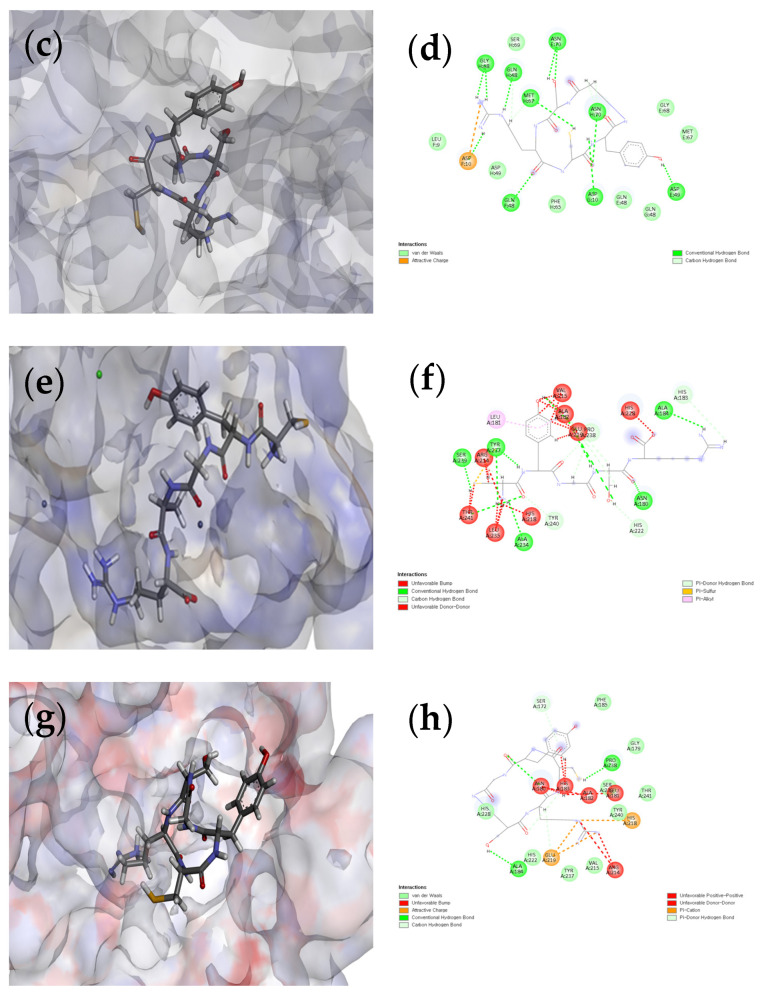
(**a**) 3D visualization of the molecular docking of L-CR5 with tyrosinase. (**b**) 2D visualization of the molecular docking of L-CR5 with tyrosinase. (**c**) 3D visualization of the molecular docking of C-CR5 with tyrosinase. (**d**) 2D visualization of the molecular docking of C-CR5 with tyrosinase. (**e**) 3D visualization of the molecular docking of L-CR5 with MMP-1. (**f**) 2D visualization of the molecular docking of L-CR5 with MMP-1. (**g**) 3D visualization of the molecular docking of C-CR5 with MMP-1. (**h**) 2D visualization of the molecular docking of C-CR5 with MMP-1.

At the tyrosinase active site, C-CR5 showed strong binding affinity by forming stable and diverse interactions compared to L-CR5. C-CR5 (Figure 6a,b) stably formed hydrogen bonds and carbon-hydrogen bonds around ASP10, GLN48, MET67, and ASN70 residues. In particular, strong electrostatic attraction was provided through the Attractive Charge interaction with the ASP10 residue, and the LEU9 and PHE65 residues secured dense packing in the hydrophobic pocket through the Van der Waals interaction. On the other hand, despite the formation of hydrogen bonds with similar residues, L-CR5 (Figure 6c,d) and salt bridge formation occurred simultaneously at the ASP10 residue. This repulsion interaction suggests that an optimal docking posture was not formed due to the flexibility of the peptide, and L-CR5 acted as a major factor explaining the relatively low MolDock score. In conclusion, unlike L-CR5, C-CR5 forms more hydrogen bonds with stable Attractive Charge without repulsion at ASP residues, demonstrating that the cyclization of peptides significantly improved their structural fitness for tyrosinase active sites.

In the docking result with MMP-1, C-CR5 also showed superior bonding stability compared to L-CR5. C-CR5 (Figure 6e,f) formed a stable hydrogen bond with ASN180, ALA184, and PRO238 near the active site. However, unfavorable interactions such as stereoscopic collision were observed at some residues of C-CR5. This interaction seems to be caused by the active site structure geometric constraints, and although the bonding direction of C-CR5 is not in a completely optimized state, an attractive charge interaction appeared around HIS218 and GLU219, contributing greatly to the reduction in binding energy. In addition, L-CR5 (Figure 6g,h) formed a hydrogen bond with the residues ASN180, ALA184, ALA234, TYR237, and SER239, but stereoscopic collision interactions occurred at multiple residues. This extensive structural collision means that L-CR5 did not form an optimal posture with the active site and acted as the main cause of the low MolDock score. Overall, although C-CR5 includes some unfavorable interactions, these interactions are limited and the overall interaction network is stable, whereas L-CR5 exhibits extensive unfavorable interactions and leads to structural instability, which is considered to have a greater impact on binding affinity.

## 4. Discussion

The enhanced antioxidant activity, tyrosinase inhibitory effect, and MMP-1 suppression observed for C-CR5 in this study suggest that this cyclic peptide can positively modulate multiple biological pathways associated with skin protection. Compared with L-CR5, C-CR5 exhibited more stable binding behaviors in molecular docking analyses, indicating that the structural constraints induced by cyclization may favorably regulate interactions with target proteins. These findings extend beyond a simple comparison of in vitro activities and support the potential of C-CR5 as a functional peptide candidate for pharmacological or cosmetic applications aimed at skin protection.

Furthermore, the present results demonstrate that short peptide sequences, such as CR5, can simultaneously achieve structural stability and enhanced biological activity through head-to-tail cyclization without the use of side-chain protecting groups. This observation provides meaningful insights into peptide design strategies, highlighting cyclization as an effective structural approach for improving the functional performance of short peptides while maintaining synthetic practicality.

It has been reported that C-CR5 has superior efficacy compared to several other studies in the existing literature [15]. In our previous study, C-CR5 was reported as a promising multifunctional peptide, and the present study builds upon this finding by directly comparing its biological performance with that of the linear form, L-CR5. The present study provides a direct comparative case that linear cyclization using a known C-CR5 markedly improves the biological performance. Although linear peptides have the advantageous flexibility for synthesis and sequence design, these structural degrees of freedom often lead to poor metabolic stability and reduced biological efficacy. The results of the present study clearly show that cyclization has the potential to address this limitation. In the DPPH radical scavenging ability assessment of linearity and ring, C-CR5 (IC_50_ = 16.92 ± 0.47 μg/mL (29.86 ± 0.83 μM)) showed significantly higher antioxidant capacity with an IC_50_ value approximately 22 times lower than that of L-CR5 (IC_50_ = 37.83 ± 34.32 μg/mL (646.25 ± 58.70 μM)) indicating a dramatic improvement in radical scavenging efficiency, and this enhancement anticipates the potential for more effective electron donating and radical stabilization due to structural locking of Tyr and Cys residues. Likewise, tyrosinase inhibition and MMP-1 inhibition were consistently superior in the cyclic form. In terms of tyrosinase inhibition, IC50 of C-CR5 (IC_50_ = 104.2 ± 5.3 μg/mL (184.0 ± 9.4 μm) showed 6.5 times more inhibitory activity than L-CR5 (IC_50_ = 680.63 ± 15.68 μg/mL (1164.0 ± 27.3 μm)), and C-CR5 showed inhibitory activity close to twice that of L-CR5 at the same concentration. When evaluating MMP-1 expression, L-CR5 did not show a tendency to decrease expression, but C-CR5 showed a decrease in expression by about 93%, reflecting that the structural complementarity with the enzyme active site was improved.

As a result of molecular docking, C-CR5 showed a lower MolDock score and hydrogen bond compared to L-CR5 in both tyrosinase and MMP-1. This suggests a certain level of structural consistency with the experimentally confirmed superiority of physiological activity. In the molecular interaction analysis, the difference in binding strength between the two peptides could be explained by the difference in structural rigidity. In the case of tyrosinase, cyclic C-CR5 showed a pose that maintained high structural suitability with the active site by forming a stable attractive charge interaction and multiple hydrogen bonds. On the other hand, unfavorable negative–negative repulsion interactions were observed at some residues of the linear L-CR5 due to its high flexibility, and this interaction is considered to be a likely factor to decrease the stability of the binding pose. A similar trend was observed in MMP-1. In the L-CR5, a number of stereoscopic collisions were observed in the docking pose, suggesting the possibility of limiting the formation of an optimal binding posture as the structural flexibility does not completely match the shape of the active site. As a result, it is interpreted that the MolDock score was relatively elevated, and there were some unfavorable interactions in C-CR5, but C-CR5 contributed to the increase in overall binding stability by securing strong attractive charge interactions with HIS218 and GLU219 through structural constraints. The MVD program used in this study is known to predict high affinity binding energy to a specific target, and considering that previously reported MolDock Score (−78.66, 83.85 kcal/mol), which achieved high affinity, the docking scores of L-CR5 and C-CR5 were within the excellent range [44,45].

This study is a supportive result for literature in which peptides of the same sequence have linear to cyclization conversion enhanced biological efficacy [46,47]. C-CR5 outperformed L-CR5 in all evaluated activities, including antioxidant, melanogenesis, and wrinkle prevention, and showed a more favorable molecular interaction with tyrosinase and MMP-1. The integration of in vitro assays and docking assays provides a clear structure–activity basis for the superior performance of the cyclic form. Overall, our results highlight that cyclic CYGSR is a promising multifunctional peptide with potential applications in dermatological and cosmetic formulations, and highlight the need for further SAR-based optimization of cyclic peptides.

In this study, the characteristics of linear and cyclic peptides were compared primarily based on molecular weight analysis for confirmation of successful synthesis and in vitro physiological activity evaluations. However, for a more detailed structural comparison and a clearer elucidation of the SAR, additional structural analyses are required. In particular, to better understand how subtle conformational changes induced by cyclization influence biological activity, further studies incorporating NMR analysis will be necessary in future work.

It is expected that future studies employing molecular dynamics-based analyses will enable a more in-depth elucidation of the molecular mechanisms underlying the superior biological activities of the cyclic peptide observed in this study.

This study confirmed that cyclization gave functional excellence by comparing the biological activities of L-CR5 and C-CR5 with the same sequence, but there are several limitations. First, since most of the analysis was performed at the in vitro level, in this study, in vivo characteristics such as stability, permeability, and efficacy persistence in the actual skin environment could not be directly confirmed. Second, although it is clear that the cyclization of CR5 contributed to the improvement of activity, specific structure–activity correlation (SAR), that is, changes in stereo structure due to cyclization or changes in the orientation of binding motifs, etc., could not be identified at the molecular level. Third, the molecular docking analysis is based on a static model and acts as a limitation as it is limited to fully reflect the dynamic changes in actual enzyme and peptide bonds. Finally, the linear and cyclic comparisons are limited to the same sequence, CYGSR, and further peptide library studies need to be accumulated in order to draw generalized conclusions over different sequence ranges. Despite these limitations, the results of this study that the cyclization of CR5 significantly improves the functional excellence provide useful basic data for future functional peptide design.

## 5. Conclusions

In conclusion, this study systematically compared the biological activities and molecular docking characteristics of L-CR5 and C-CR5 peptides, demonstrating that cyclization serves as a key structural factor for enhancing the functional activity of short peptides. C-CR5 exhibited superior antioxidant, whitening-related, and anti-wrinkle-associated activities, suggesting its potential utility in pharmacological and cosmetic applications for skin protection. Moreover, this work illustrates that the cyclization of short, unprotected peptides represents a practical and effective strategy for achieving both structural stability and biological efficacy, providing a useful foundation for the future development and design of functional peptide-based materials.

Existing studies have remained at the level of evaluating the single function of linear or cyclic peptides, but it is difficult to find studies that have integrated the three functions of antioxidant, whitening, and anti-wrinkle by directly comparing linear and cyclic peptides of the same sequence. In particular, quantitative comparisons based on activity increase multiples are limited in previous studies, so this study is highly likely to be utilized in that it is the first study to clearly present the structure–activity correlation according to CR5 cyclization.

## Figures and Tables

**Table 1:** Molecular Docking Results Comparing Linear and Cyclic peptides with Target Protein (Tyrosinase, MMP-1).

Peptide	Target Protein	MolDock Score (kcal/mol)	Rerank Score	H-Bond (kcal/mol)
L-CR5	Tyrosinase	−75.3578	−65.8698	−10.5355
C-CR5	Tyrosinase	−110.023	−72.9746	−14.8095
L-CR5	MMP-1	−60.0822	21.1785	−3.24147
C-CR5	MMP-1	−99.6438	27.7123	−9.19535

## Data Availability

The data presented in this study are available on request from the corresponding author. The data are not publicly available due to confidentiality and ongoing further development.

## Supplementary Materials

The following supporting information can be downloaded at: https://www.mdpi.com/article/10.3390/biom16010126/s1, Supplementary Figure S1. MALDI-TOF MS spectrum of C-CR5. The X-axis represents the mass-to-charge ratio (*m*/*z*), and the Y-axis indicates the relative ion intensity. Supplementary Figure S2. Control of DPPH radical scavenging activity, ascorbic acid (IC_50_: 6.38 ± 0.23 μg/mL (36.2 ± 1.3 μM, *n* = 3). Supplementary Figure S3. Control β-arbutin of tyrosinase inhibitory activity (IC_50_: 222.33 ± 4.5 μg/mL (816.7 ± 16.5 μM, *n* = 3)). Supplementary Figure S4. Chromatogram obtained before and after purification of L-CR5. Supplementary Figure S5. (a) Cyclic peptides (free, unbound form), (b) Linear peptides (free, unbound form) Color notation: carbon skeleton is black, oxygen/carbonyl group is red, yellow is yellow is yellow, nitrogen is blue.

## Abbreviations

C-CR5	Cyclic-CYGSR
CR5	Cys-Tyr-Gly-Ser-Arg;CYGSR
CTC	2-Cholorotrityl chloride resin
DCM	dichloro methane
DIEA	*N*,*N*-diisopropylethylamine
DIC	*N*,*N*-diisoepropylcarbodiimide
DMEM	Dulbecco’s Modified Eagle’s medium
DMF	*N*,*N*-Dimethylformamide
DPBS	Dulbecco’s Phosphate Buffered Saline
DPPH	2,2-diphenyl-1-picrylhydrazyl
EDC	*N*-ethyl-*N*′-(3-dimethylaminopropyl) carbodiimide
FBS	fetal bovine serum
HOBt	*N*-hyroxybenzotriazole
HPLC	High Performance Liquid Chromatography
IMDM	Iscove’s Modified Dulbecco’s medium
IPE	isopropyl ether
L-CR5	Linear-CYGSR
MALDI-TOF	Matrix-assisted laser desorption/ionization time-of-flight
MMP-1	Matrix Metalloproteinase-1
MVD	Molegro Virtual Docker
PDA	Photodiode array
SAR	structure-activity relationship
SPPS	solid-phase peptide synthesis
TFA	trifluoroacetic acid
TIS	triisopropylsilane
