# Supplementary material for: Comparison of In Vitro Multiple Physiological Activities of Cys–Tyr–Gly–Ser–Arg (CYGSR) Linear and Cyclic Peptides and Analysis Based on Molecular Docking
Source: Biomolecules. 2026 Jan 12;16(1):126. doi: 10.3390/biom16010126 (PMC12839123; doi:10.3390/biom16010126)
Supplement: Supplementary file 1 [file biomolecules-16-00126-s001.zip › biomolecules-4046401-supplementary.pdf]

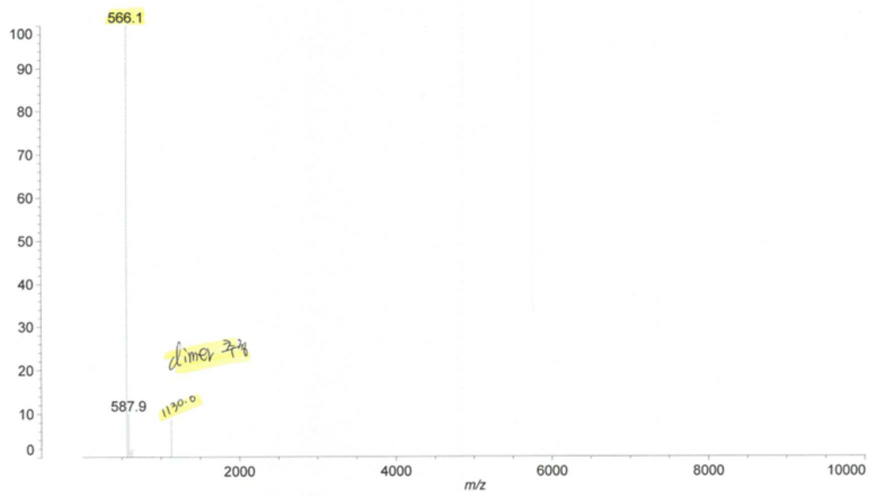

Supplementary Figure S1. MALDI-TOF MS spectrum of C-CR5. The X-axis represents the mass-to-charge ratio (m/z), and the Y-axis indicates the relative ion intensity.

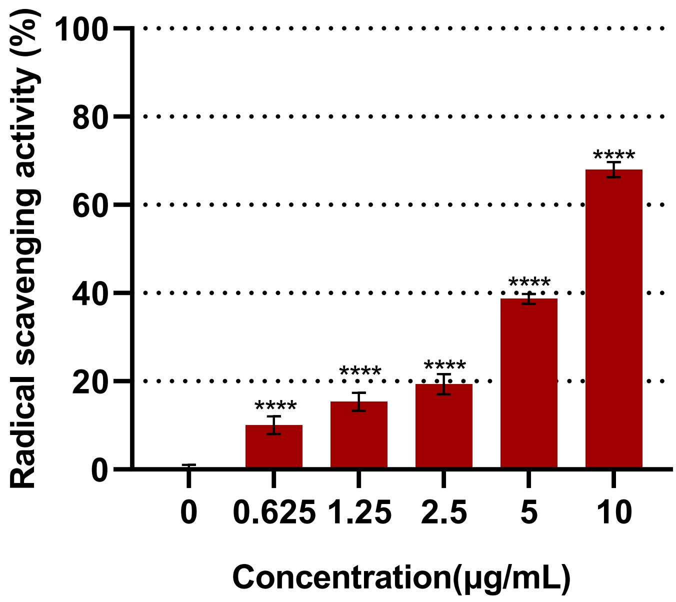

Supplementary Figure S2. Control of DPPH radical scavenging activity, ascorbic acid (IC_{50}: 6.38±0.23 μg/mL (36.2±1.3 μM, n=3).

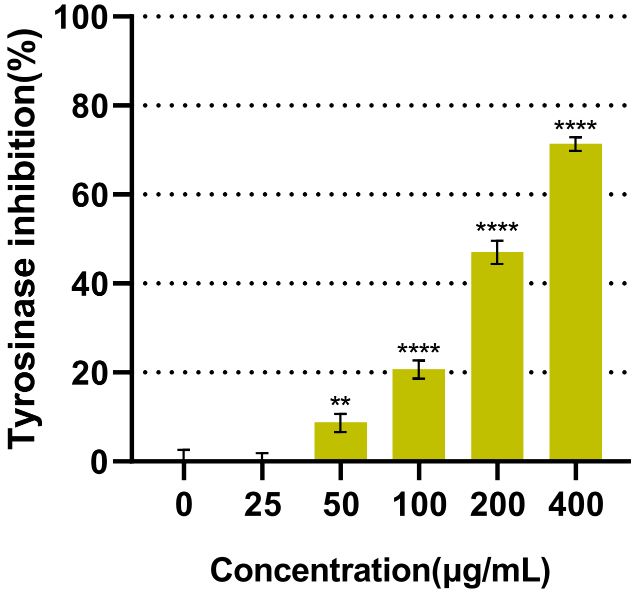

Supplementary Figure S3. Control β-arbutin of tyrosinase inhibitory activity (IC $_{50}$: 222.33±4.5 µg/mL (816.7±16.5 µM, n=3)).

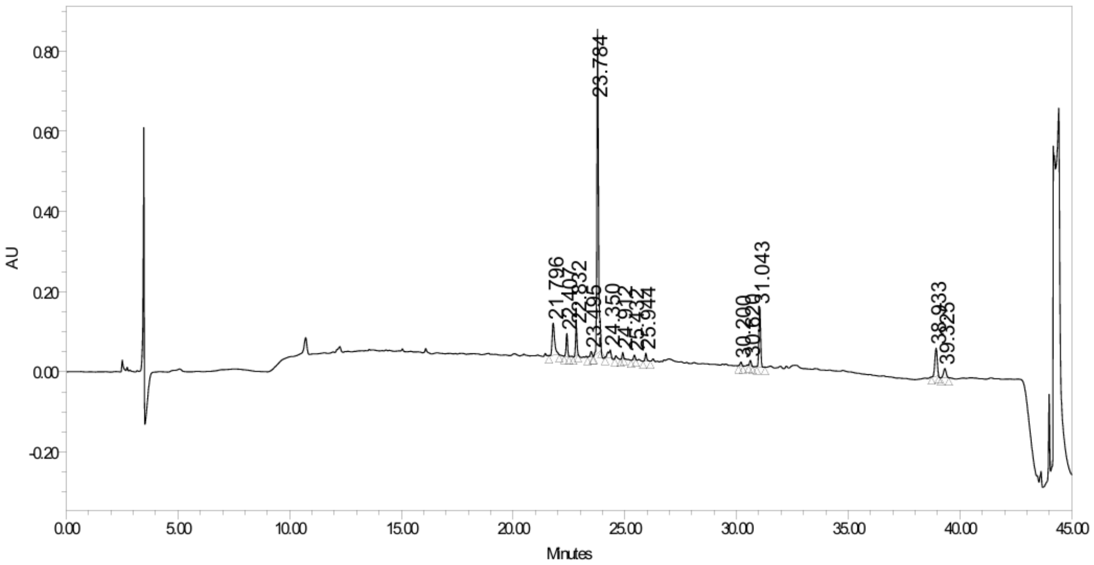

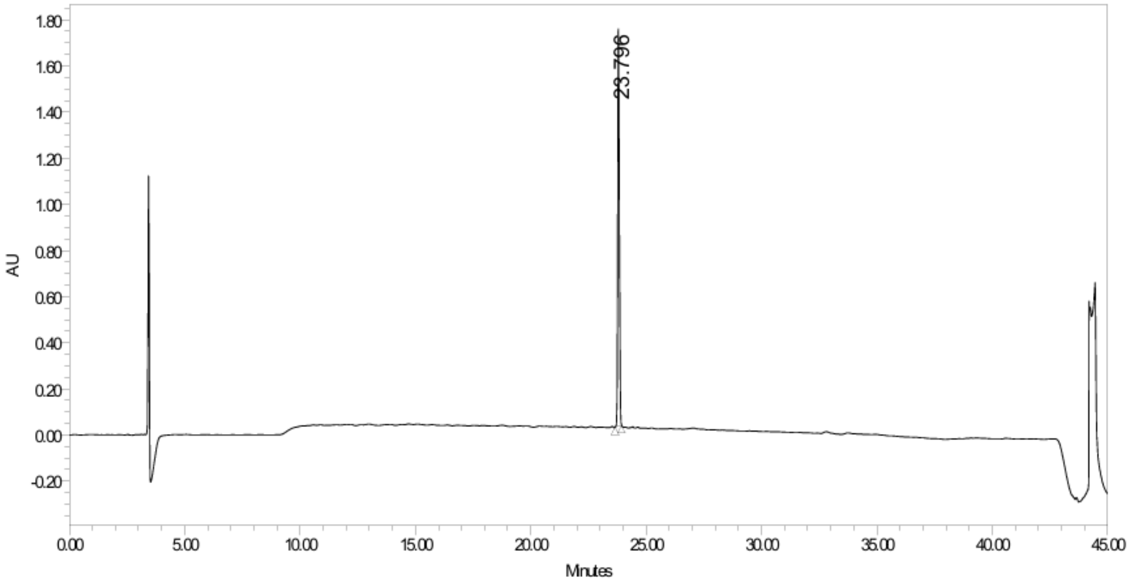

Supplementary Figure S4. Chromatogram obtained before and after purification of L-CR5

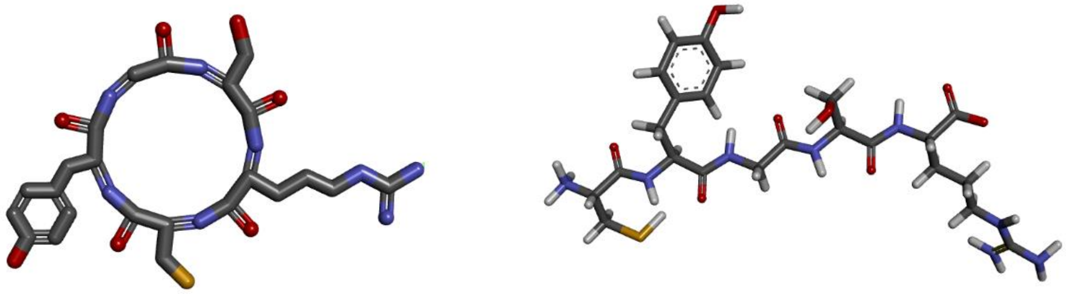

(a) (b)

Supplementary Figure S5. Color notation: carbon skeleton is black, oxygen/carbonyl group is red, yellow is yellow is yellow, nitrogen is blue
